# Nanosecond Pulsed Electric Field Inhibits Cancer Growth Followed by Alteration in Expressions of NF-κB and Wnt/β-Catenin Signaling Molecules

**DOI:** 10.1371/journal.pone.0074322

**Published:** 2013-09-17

**Authors:** Zhigang Ren, Xinhua Chen, Guangying Cui, Shengyong Yin, Luyan Chen, Jianwen Jiang, Zhenhua Hu, Haiyang Xie, Shusen Zheng, Lin Zhou

**Affiliations:** 1 Key Laboratory of Combined Multi-organ Transplantation, Ministry of Public Health, First Affiliated Hospital, School of Medicine, Zhejiang University, Hangzhou, Zhejiang Province, China; 2 Department of Hepatobiliary and Pancreatic Surgery, First Affiliated Hospital, School of Medicine, Zhejiang University, Hangzhou, Zhejiang Province, China; 3 State Key Laboratory for Diagnosis and Treatment of Infectious Disease, First Affiliated Hospital, School of Medicine, Zhejiang University, Hangzhou, Zhejiang Province, China; Florida International University, United States of America

## Abstract

Cancer remains a leading cause of death worldwide and total number of cases globally is increasing. Novel treatment strategies are therefore desperately required for radical treatment of cancers and long survival of patients. A new technology using high pulsed electric field has emerged from military application into biology and medicine by applying nsPEF as a means to inhibit cancer. However, molecular mechanisms of nsPEF on tumors or cancers are still unclear. In this paper, we found that nsPEF had extensive biological effects in cancers, and clarified its possible molecular mechanisms *in vitro* and *in vivo*. It could not only induce cell apoptosis via dependent-mitochondria intrinsic apoptosis pathway that was triggered by imbalance of anti- or pro-apoptosis Bcl-2 family proteins, but also inhibit cell proliferation through repressing NF-κB signaling pathway to reduce expressions of cyclin proteins. Moreover, nsPEF could also inactivate metastasis and invasion in cancer cells by suppressing Wnt/β-Catenin signaling pathway to down-regulating expressions of VEGF and MMPs family proteins. More importantly, nsPEF could function safely and effectively as an anti-cancer therapy through inducing tumor cell apoptosis, destroying tumor microenvironment, and depressing angiogenesis in tumor tissue *in vivo*. These findings may provide a creative and effective therapeutic strategy for cancers.

## Introduction

Cancer are still a leading cause of death worldwide and accounted for 7.6 million deaths (around 13% of all deaths) in 2008 according to WHO, and deaths from cancer are projected to rise to over 11 million in 2030 [1]. A significant proportion of cancers can be cured by surgery, radiotherapy or chemotherapy, especially if they are detected early. However, some kinds of cancers cannot be discovered in early stage and have little response to radiotherapy and chemotherapy, thus patients with such cancers have a poor prognosis. For example, pancreatic cancer has approximately 23% 1-year survival after diagnosis and 5% 5-year survival at best [2]. Furthermore, there were an estimated 43,140 new cases and 36, 800 deaths attributed to pancreatic cancer in the United States [3,4]. It is essential to search for a new therapy technique to improve prognosis and survival of cancer patients.

A new technology using high pulsed electric field has emerged from military application into biology and medicine by applying nanosecond pulse electric field (nsPEF) as a means to inhibit cancer [5]. The main characteristic of nsPEF is its high power and low energy leading to very little heat production and its special ability to penetrate into cell to influence intracellular organelles [6,7]. Quite different from classical plasma membrane electroporation, nsPEF can produce highly compressed power (billions of watts), ultra short pulse durations (nanosecond), rapid rise times (nanosecond) and high electric fields (kV/cm). The resulting nanosecond pulse can penetrate into cell before plasma membrane is fully charged allowing nsPEF to have minimal affect plasma membrane therefore not causing electroporation [8]. Recent years, studies have been done to determine effects of nsPEF in experimental and model researches. The results proved that nsPEF could induce apoptosis in various cancer cell lines *in vitro* [9,10] and a fibrosarcoma tumor *ex vivo* [7], and eradicate B16f10 melanoma tumor *in vivo* [11,12,13]. However, molecular mechanisms of biological effects of nsPEF on tumors or cancers are still unclear.

In this research, we sought to investigate anti-cancer effect of nsPEF and its possible molecular mechanisms through *in vitro* and *in vivo* experiments. Here, we showed that nsPEF could significantly inhibit cancer growth *in vitro* and *in vivo* via inducing apoptosis, inhibiting proliferation, inactivating invasion and metastasis, and destroying tumor microenvironment, which will provide a novel and effective therapeutic strategy for cancers.

## Materials and Methods

### Cell culture

Human pancreatic carcinoma cell line (PANC-1) and HCC cell line (Hep-3B) were purchased from Cell Bank of Chinese Academy of Science (Shanghai, China). Both cell lines were cultured in Dulbecco’s modified Eagle medium (DMEM, Gibco-Invitrogen, Carlsbad, CA, USA) supplemented with 10% fetal bovine serum (FBS, SAFC Biosciences, Lenexa, KS, USA), 100 units/mL penicillin and 100 mg/mL streptomycin (Sigma-Aldrich, St. Louis, MO, USA).

### Induction of cell death by nsPEF

As our previous description [11], nsPEF generator with duration of 100-ns was shown in Figure **S1**. Electric fields varied from 20kV/cm to 60kV/cm. Waveforms were monitored with a digital phosphor oscilloscope (Figure S1 A& B, DPO4054, Tektronix, USA) equipped with a high voltage probe (P6015A, Tektronix, USA). PANC-1 cells were harvested with trypsin and re-suspended in fresh DMEM medium with 10% FBS to a concentration of 5.0×10^6^ cells/ml. 500µl of cell suspension were placed into a 0.1cm gap cuvette (Figure **S1 C**, Biosmith, aluminum plate electrodes) and exposed to 100 pulses at 0, 20, 40 and 60 kV/cm electric field strength respectively. Most of detections of cell responses were performed at 1h after treatment, mainly including transwell assay, cell TEM, DNA ladder assay, cell TUNEL assay, flow cytometry and western-blot. Cell viability and proliferative inhibition rate were measured at different time points after treatment to observe a gradual active process. The whole experiments were repeated for three times.

### Measurement of cell viability and proliferative inhibition rate

PANC-1 cells were exposed to nsPEF and then cultured. 2×10^5^ cells were exposed to nsPEF with different intensities, and then cultured for 0, 0.5, 1, 2, 24 and 48 h respectively. The cells were trypsinized and viable cells were counted by a cell viability analyzer (Vi-cell, Backman). After incubation for 24, 48 and 72 h respectively, cells were calculated by Cell Counting Kit-8 (CCK-8) assay (Dojindo Laboratories, Kumamoto, Japan) according to manufacturer’s instructions, reflecting cell proliferative inhibition.

### Detection of cell metastasis and invasion ability with transwell assay

At 1 h after nsPEF treatment, the treated survival cells at the same number were obtained to perform transwell assays based on transwell chambers (Millipore, USA), reflecting cell metastasis and invasion ability, as previously described [14].

### Observation of cell ultrastructure by TEM

At 1 h after nsPEF treatment, the treated cells were obtained and fixed with 2.5% glutaraldehyde to observe cell ultrastructure by transmission electron microscopy (TEM) in Imaging Facility of Core Facilities, Zhejiang University School of Medicine, as previously described [15].

### Determination of DNA fragmentation with DNA ladder assay

At 1 h after nsPEF treatment, the treated cells were obtained to investigate cell DNA fragmentation by DNA ladder assay according to manufacturer’s instruction as previously described [11].

### Measurement of single-cell apoptosis with TUNEL assay

At 1 h after nsPEF treatment, the treated cells were obtained to determine single-cell apoptosis using the assay of TdT–dUTP Terminal Nick-end Labeling (TUNEL) with *In Situ* Cell Death Detection Kit (Millipore, USA) according to manufacturer’s instruction, as previously described [14].

### Detection of cell apoptosis with flow cytometry

At 1 h after nsPEF treatment, the treated cells were obtained to detect cell apoptosis by Annexin V-FITC Apoptosis Detection Kit (BD Biosciences) as previously described [16].

### Analysis of cell cycle with flow cytometry

At 1 h after nsPEF treatment, the treated cells were obtained to determine cell cycle change. Cell cycle assay and analysis was carried out as previously described [17].

### Western blotting analysis

At 1 h after nsPEF treatment, the treated cells were obtained to extract protein. Protein extraction and Western blotting analysis were performed as previously described [18]. Primary antibodies and details are listed in Table **S1**.

### Tumor induction in nude mice with Hep-3B cells

The 5 weeks old nude mice were purchased from Shanghai Experimental Animal Centre, Chinese Academy of Science. Experimental animals were kept in the central animal facility of Zhejiang University School of Medicine and housed in laminar-ﬂow cabinets under speciﬁc pathogen-free conditions at 22 °C with a 12 h light/dark cycle. All mice were anesthetized with ketamine (100 mg/kg intraperitoneal). Tumor-bearing mice models were established through injection of 2×10^6^ Hep-3B cells in the right abdomen in mice. At 1 week after cell injection, tumor was typically 3 mm wide and had exhibited angiogenesis. When tumors grew to 10 mm wide or more--- typically about 4 weeks after cell injection, 50 tumor-bearing mice models were successfully established and divided into two groups: Control group (n=17) and nsPEF group (n=33). The mice in the nsPEF group were exposed to nsPEF (Figure **S1 D& E**, 100 pulses, 20kV/cm) with the clamp. The clamp electrodes were made from plastic holder clamp with 5-mm wide thin copper to cover the tumor. We coated these electrodes with an ultrasound gel to separate the skin from the electrode. For treatment, each tumor was positioned between two palms of the clamps with a separation of 0.5 mm, while 100 ns pulses in duration and 20 kV/cm in intensity were applied at a frequency of 0.5 Hz. Throughout the experiment, the mouse was anesthetized and positioned on a warming stage that maintains the mouse’s body temperature within the normal range. Physical activities and weights of the mice as well as each tumor were observed and recorded once or twice per week. All procedures were performed in accordance with the ‘‘Guide for the Care and Use of Laboratory Animals’’ published by the National Institutes of Health (NIH publication 86–23 revised 1985). Animal protocols were approved by Animal Care and Facilities Committee of Zhejiang University.

### Tumor *in vivo* imaging with special MRI of mouse

Tumor-bearing mice from Control group and nsPEF group were anesthetized and put into the small coil for mouse (Peking University, China), and then placed into NMR machine (Advance 400, Hruker Company, Swiss). The *in vivo* image of mice with tumor was taken.

### Sample collection

On about 6.5 weeks after cell injection, all mice were sacrificed by over-dosed anesthesia and all tumors were dissected to compare. Tumor tissue was fixed into Phosphate-buffered formalin (10%) to perform H&E staining, TUNEL assay, and IHC staining. Meanwhile, part of tumor was fixed into 2.5% glutaraldehyde (4°C, pH 7.4) to perform tumor TEM.

### H&E staining and Immunohistochemistry

H&E staining and Immunohistochemistry (IHC) were performed as we have previously described [19]. Primary antibodies and details are listed in Table **S1**.

### Tumor in situ detection of apoptosis with TUNEL assay

Tumor cell apoptosis was measured using TUNEL assay, carried out as previously described [14].

### Statistical analysis

The present data are expressed as mean ± SEM. Graph Pad Prism 5 software was applied for statistical analysis. Cell apoptosis and cell cycle were analyzed by FlowJo 7.6.1 Software. Relative intensity of each protein band was analyzed by Photoshop CS4 software. IHC results were analyzed by Image Pro-Plus software. One-way analysis of variance (ANOVA) with Dunnett’s multiple comparisons and an unpaired, two-tailed Student’s t test were used for data analysis. Statistical significance was set at P value < 0.05.

## Results and Discussion

Despite intensive efforts in cancers treatment practices, survival rates for some kinds of cancers have not been substantially improved in past years, such as pancreatic cancer [2], prostatic carcinoma [20] and lung cancer [21]. Novel treatment strategies are therefore desperately required for treatment of cancers and long survival of patients. During the multistep development, human cancers acquire ten biological hallmarks including resisting cell death, sustaining proliferative signaling, activating invasion and metastasis, enabling replicative immortality, evading growth suppressors, inducing angiogenesis, reprogramming of energy metabolism, evading immune destruction, tumor-promoting inflammation, and genome instability and mutation [22,23]. These hallmarks constitute an organizing principle for rationalizing the complexities of neoplastic disease and also become major targets for cancer research and therapeutic strategies. As a relatively new technology to interpret cancer, nsPEF is effective in various cell lines *in vitro* [5,9,10], and B16f10 melanoma and hepatocellular carcinoma [11,24], as well as human pancreatic carcinoma [25] *in vivo*, demonstrating potential application prospect of nsPEF for cancer therapy. However, until now, it remains unclear on molecular mechanisms of nsPEF on cancers.

In this study, we used cancer models both *in vitro* and *in vivo* experiments to search for possible molecular mechanisms.

### NsPEF induced cancer cells death and proliferative inhibition *in vitro*

**Figure 1:**
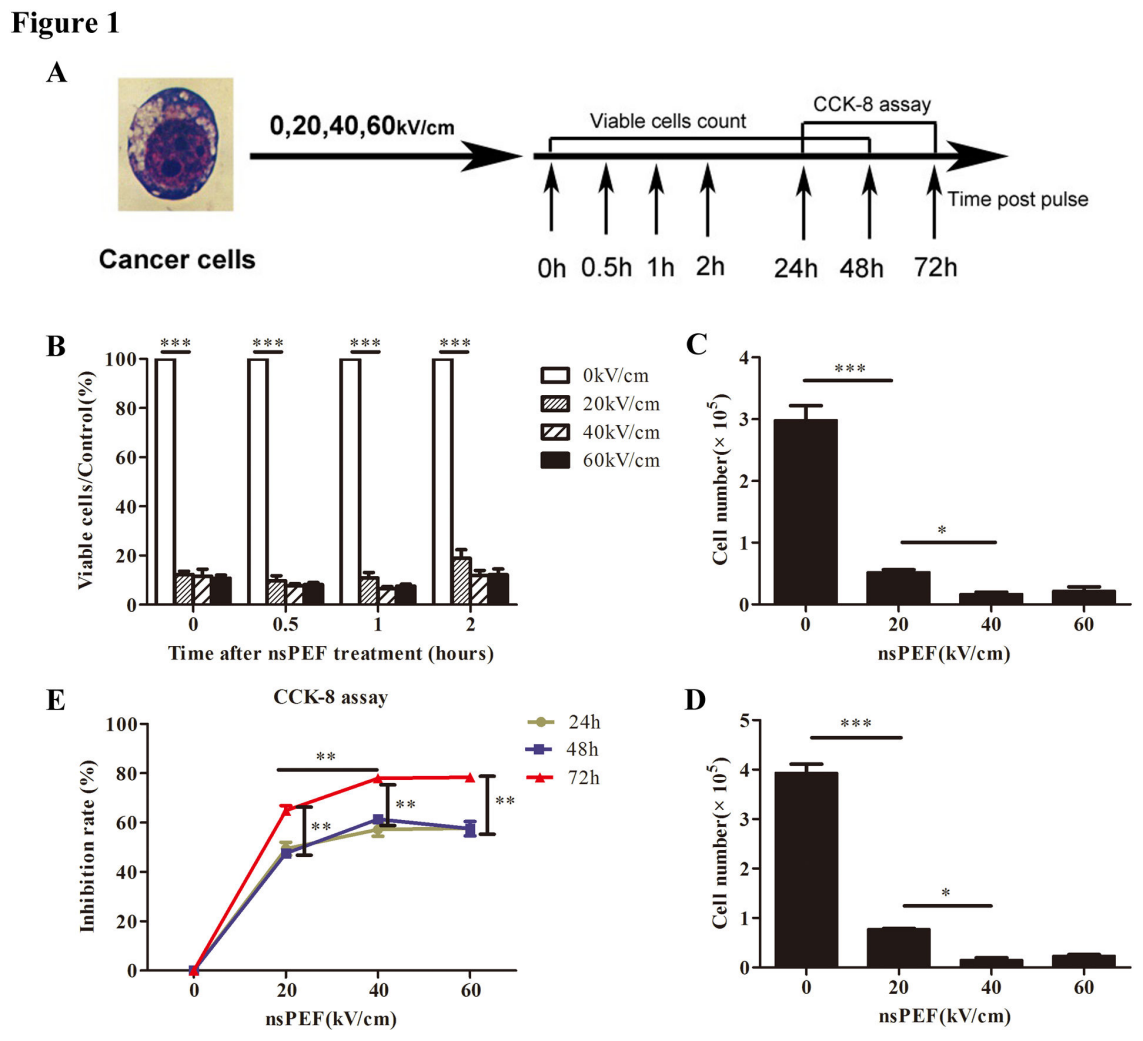
NsPEF induces cancer cells death and proliferative inhibition *in vitro.* (A) Schedule of cancer cells exposed to nsPEF with different intensities for viable cells count and CCK-8 assay. (B) The rate of viable cells/control was detected by counting trypan blue negative cells at 0 h, 0.5 h, 1 h, and 2 h post pulses with different intensities. ***p<0.001. (C and D) Numbers of viable cells were calculated after exposure to nsPEF with different intensities for 24 h and 48 h respectively. *p<0.05, ***p<0.001. (E) According to CCK-8 assay, inhibition rates of cells proliferation induced by nsPEF with different intensities were detected. **p<0.01.

To address effect of nsPEF in PANC-1 cells, cells were exposed to nsPEF with electric field at 0, 20, 40 and 60 kV/cm respectively (Figure **1A**). The rate of viable cells/control was detected by counting trypan blue negative cells at 0h, 0.5h, 1h and 2h post pulse (Figure **1B**). We found that viable cells post pulse were significantly decreased compared with the control (p<0.001), but the time-dependent manner and dose-dependent manner of this reduced trend were not observed in a short time post pulse, at least within 2h. Moreover, treated cells were cultured for 24h and 48h, and then calculated respectively. We found that the viable cells were also remarkably reduced versus the control (both p<0.001) (Figure **1C& D**). In addition, inhibition rates of cell proliferation, detected by CCK-8 assay, were remarkably increasing in different intensities at 48h and 72h after exposure to nsPEF. Especially at 72h after pulse, higher intensities acquired higher inhibition rates, which displayed a dose-dependent manner, indicating that the proliferation of survival cells was influenced with dose-dependence (Figure **1E**). Figure 1 showed not only cells deaths, but also inhibition of cell proliferation induced by nsPEF.

### NsPEF induced cell apoptosis via the dependent-mitochondria intrinsic apoptotic pathway *in vitro*

**Figure 2:**
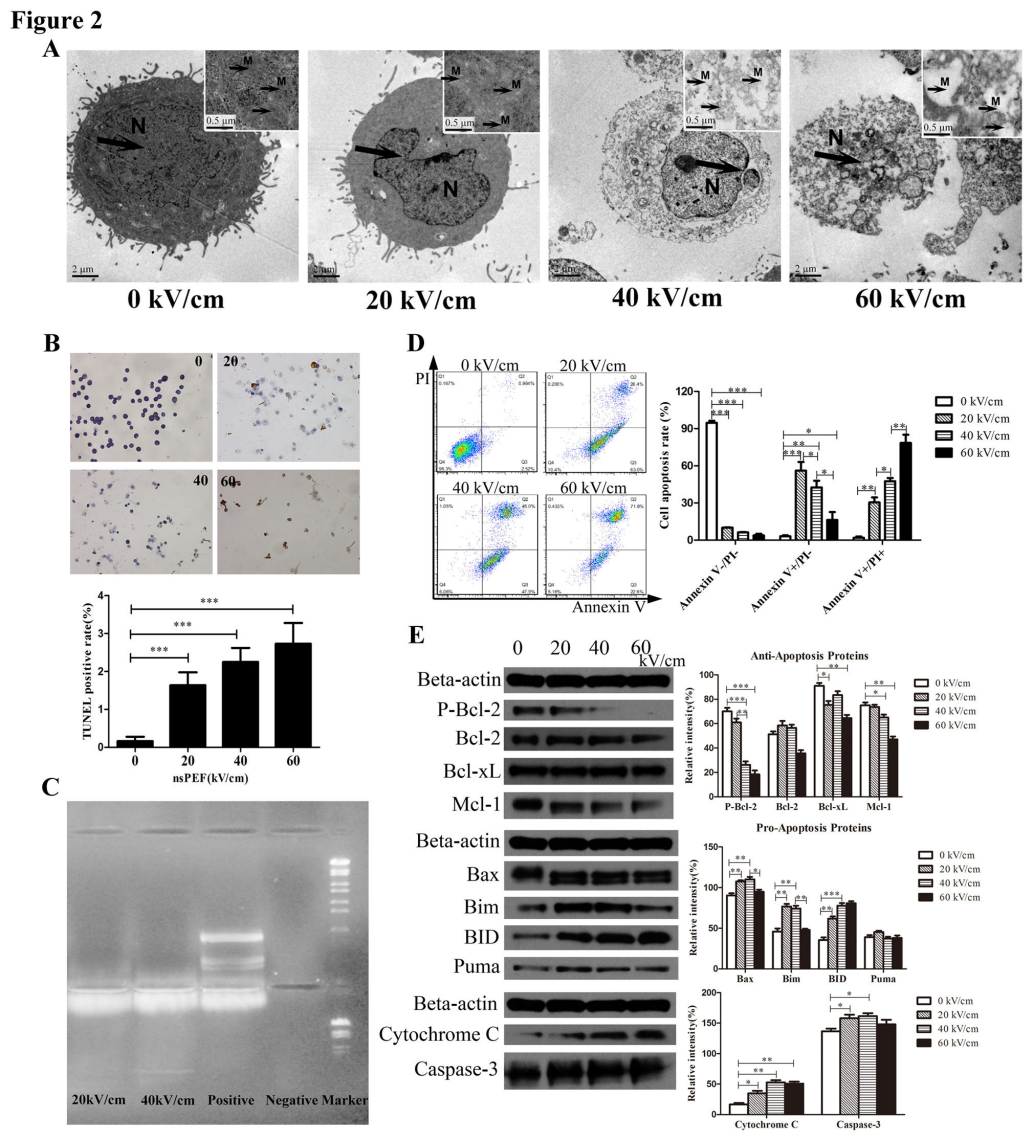
NsPEF induces cancer cells apoptosis via dependent-mitochondria intrinsic apoptotic pathway *in vitro.* (A) Morphology changes of cancer cells exposed to nsPEF with different intensities were observed by cell transmission electron microscopy (TEM). N: nuclear changes including nuclear shrinkage, nuclear membrane blebbing and nuclear lysis. M: mitochondria degeneration or vacuolar degeneration. (B) Single cell apoptosis of cancer cells exposed to nsPEF with different intensities was calculated by cell TUNEL assay. Original magnification, 400×. ***p<0.001. (C) Cell DNA fragmentation of cancer cells exposed to nsPEF was observed by apoptosis DNA ladder assay. (D) Apoptosis rates of cancer cells exposed to nsPEF with different intensities were tested with PI and Annexin-V staining assay by flow cytometry. Results were analyzed by FlowJo 7.6.1 Software. *p<0.05, **p<0.01, ***p<0.001. (E) Protein expressions of anti-/pro-apoptosis Bcl-2 family, Cytochrome C and Caspase-3 in cancer cells after exposure to nsPEF with different intensities were detected by Western-blot assay. Relative intensity of each protein was analyzed by Photoshop CS4 software. *p<0.05, **p<0.01, ***p<0.001.

First, to investigate death characteristics induced by nsPEF in pancreatic carcinoma cells, we detected morphology changes of cells after treatment by TEM (Figure **2A**). Apoptotic characteristics of cells were rarely observed in the control (0kV/cm). However, treated cells with 20kV/cm nsPEF began to present cell morphology alteration of early apoptosis: nuclear shrinkage, nuclear notch, chromatin condensation and margination, and minor mitochondria degeneration. Under effect of 40kV/cm nsPEF, further changes were observed, mainly including chromatin clumping, cytoplasm condensing and some vacuoles appearing on cell membrane or in cytoplasm. Meanwhile, nuclear fragments and cytoplasm constituents were packaged into apoptotic bodies, and mitochondria showed vacuolar degeneration. When electric fields increased up to 60kV/cm, treated cells presented membrane lysis, nuclear membrane blebbing, nuclear lysis and fragmentation as well as mitochondria damage, which were typically considered as necrosis.

Subsequently, we studied DNA damage and fragmentation by TUNEL assay and apoptosis DNA ladder assay. In TUNEL staining, the labels combined damaged sites of DNA, and apoptotic cells were stained brownish–yellow. We observed that TUNEL positive rates were obviously rising up in PANC-1 cells post pulse at 20, 40, and 60 kV/cm compared with the control, respectively (all p<0.001) (Figure **2B**). Consistent with TUNEL results, DNA fragmentation presented in PANC-1 cells exposed to nsPEF at 20 and 40 kV/cm as compared to positive control and negative control from DNA ladder assay (Figure **2C**).

To further investigate apoptotic degree in cancer cells post pulse, we tested apoptotic rates of treated cells with Annexin-V/PI staining by flow cytometry (Figure **2D**). Compared with the control, the rate of Annexin V^-^PI^-^ cells (viable cells) was significantly decreased after exposure to nsPEF, but the rate of Annexin V^+^PI^-^ cells (early apoptosis) was remarkably increased at 20kV/cm (p<0.001), and then significantly decreased at 40kV/cm (p<0.05) and 60kV/cm (p<0.01). In sharp contrast, the rate of Annexin V^+^PI^+^ cells (late apoptosis or necrosis) was continually increasing in dose-dependence at 20, 40 and 60 kV/cm (all p<0.01). These data suggested cells death induced by nsPEF presented from early apoptosis to late apoptosis or necrosis with increased intensities of nsPEF, in other words, dose-dependence. These results were identical with our findings via cell TEM.

Recent researches proved that apoptosis played an important role in cell death induced by nsPEF [11,26]. Consistent with these studies, we found apoptotic body and mitochondria degeneration by TEM after nsPEF treatment. Furthermore, results of TUNEL assay, apoptosis DNA ladder assay and Annexin-V/PI staining also proved apoptosis induced by nsPEF in PANC-1 cells. Some studies [5,9] reported that cancer cell death induced by nsPEF was mainly attributed to apoptosis, and interpreted that increase of Annexin V^+^PI^+^ cells in dose-dependence might be ascribed to a special “mimicking necrosis” that high dose pulse induced bigger holes in cell membranes and then PI stain entered cells. However, our further evidence via cell TEM proved that cell death presented from early apoptosis to late apoptosis or necrosis with increased intensities of nsPEF, not just cell apoptosis.

The intrinsic apoptosis induced by stress-inducing stimuli and extrinsic apoptosis via death receptor activation are two main pathways of apoptosis. Mitochondria function and anti-/pro-apoptosis Bcl-2 proteins family play essential roles in intrinsic apoptosis pathway [27]. Anti-/pro-apoptosis Bcl-2 proteins family members are initially integral membrane proteins found in the mitochondria, endoplasmic reticulum (ER), or nuclear membrane [28]. A major activating site of Bcl-2 proteins is mitochondrial membrane [27]. To explore possible mechanisms of apoptosis induced by nsPEF, we detected relative proteins expression of the intrinsic apoptotic pathway, such as pro-apoptosis Bcl-2 family proteins (Bad, p-Bad, Bax, Bik, Bim, BID, Bak and Puma), pro-survival Bcl-2 family proteins (p-Bcl-2, Bcl-2, Bcl-xL and Mcl-1), and apoptosis directly relative proteins (Cytochrome-C and Caspase-3) (Figure **2E**). Compared to the control, expressions of anti-apoptosis Bcl-2 family proteins, mainly including p-Bcl-2, Bcl-xL and Mcl-1, were remarkably reduced at different degrees, while expressions of pro-apoptosis Bcl-2 family proteins, mainly including Bax, Bim and BID, were significantly elevated at different degrees with increased intensities of nsPEF. Meanwhile, expressions of Cytochrome-C and Caspase-3 were obviously increased after exposure to nsPEF. We concluded that the apoptosis induced by nsPEF was triggered by regulating imbalance of anti- or pro-apoptosis Bcl-2 family proteins on the mitochondrial membrane. These results were consistent with mitochondria degeneration and damage in cell ultra-structure. Collectively, these apoptotic data indicated that nsPEF induced apoptosis in PANC-1 cells via the dependent mitochondria intrinsic apoptosis pathway that was triggered by imbalance of anti- or pro-apoptosis Bcl-2 family proteins.

### NsPEF inhibited cell proliferation via repressing NF-κB signaling pathway *in vitro*

**Figure 3:**
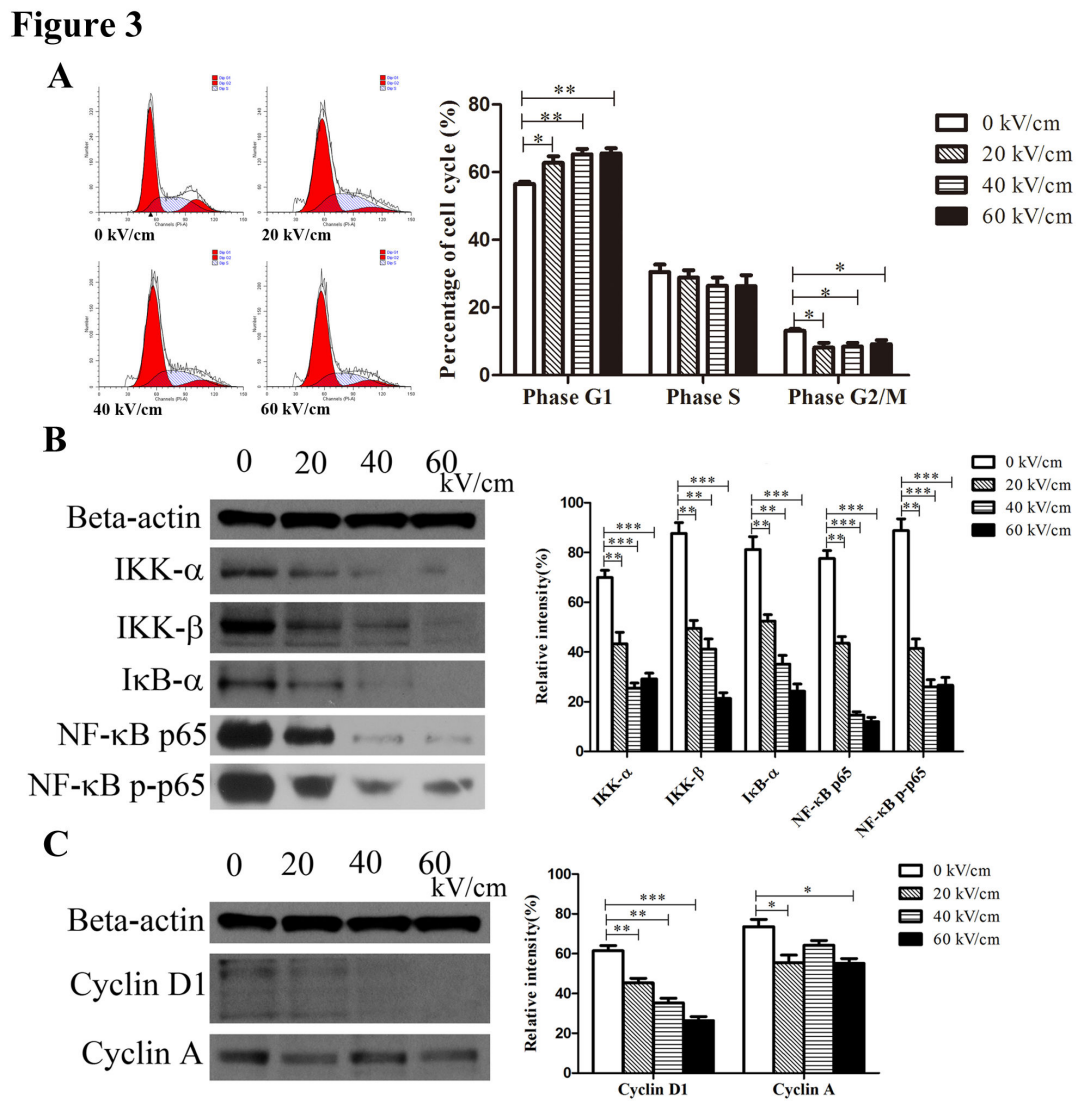
NsPEF inhibits cell proliferation through repressing NF-κB signaling pathway to reduce expressions of Cyclin proteins *in vitro.* (A) Cell cycle of cancer cells exposed to nsPEF with different intensities was examined with PI staining assay by flow cytometry, and results were analyzed by FlowJo 7.6.1 Software. *p<0.05, **p<0.01. (B) Protein expressions of NF-κB signaling pathway including IKK-α, IKK-β IκB–α, p65 and p-p65 in cancer cells after exposure to nsPEF with different intensities were detected by Western-blot assay. (C) Protein expressions of Cyclin proteins including Cyclin D1 and Cyclin A in cancer cells after exposure to nsPEF with different intensities were detected by Western-blot assay. Relative intensity of each protein was analyzed by Photoshop CS4 software. *p<0.05, **p<0.01, ***p<0.001.

Besides apoptosis, nsPEF had a remarkable influence on cell proliferation from our CCK-8 assay that inhibition rate of cell proliferation was remarkably increased in a manner of dose-dependence and time-dependence after 48h post pulse. In cell cycle, the translation from phase G1 to phase S and percentage of phase G2/M typically reflects cell proliferative ability. We examined cell cycle of treated cells, and found that the percentage of phase G1 was obviously increased (p<0.01), while percentage of phase G2/M was decreased (p<0.05) after exposure to nsPEF (Figure **3A**), indicating that nsPEF could arrest phase G1 of cell cycle to inhibit cell proliferation.

NF-κB signaling pathway plays an important role in cell proliferation [29]. Cancer growth inhibition via inhibiting NF-κB pathway has recently been reported in several human carcinomas, such as colon [30], lung [31] and breast [32]. The target genes regulating cell proliferation via NF-κB pathway include c-Myc, CyclinD1, Cyclin E and CDK2 which play an essential role in positive or negative regulation of cell cycle [33,34]. To further explore possible mechanisms of proliferative inactivity of pulsed cells, we detected expressions of NF-κB signaling pathway proteins and Cyclin proteins in PANC-1 cells. Our results revealed that expressions of NF-κB pathway proteins including IKK-α, IKK-β, IκB-α, NF-κB p-65 and p-p65 were obviously decreased after exposure to nsPEF versus the control (all p<0.01 or 0.001) (Figure **3B**). Higher intensity of nsPEF led to much lower expressions of these proteins compared with the control. Meanwhile, expressions of Cyclin proteins including CyclinD1 and Cyclin A were also significantly reduced after exposure to nsPEF versus the control (p<0.01, 0.001, or 0.05) (Figure **3C**). So we considered that nsPEF could depress NF-κB signaling pathway to reduce expressions of Cyclin proteins.

These data suggested that nsPEF inhibited cell proliferation through repressing NF-κB signaling pathway to reduce expressions of Cyclin proteins, thereby arresting phase G1 of cell cycle.

### NsPEF inactivated cell migration and invasion via suppressing Wnt/β-Catenin signaling pathway *in vitro*

**Figure 4:**
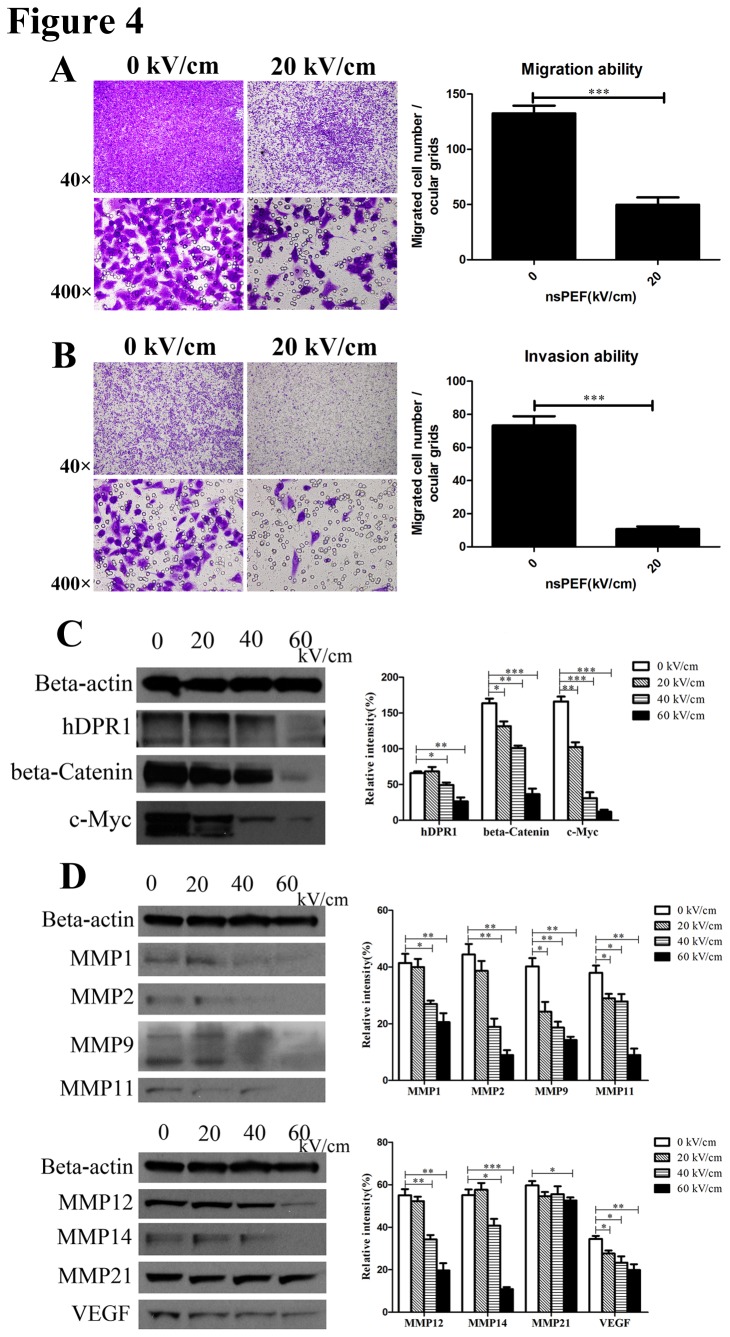
NsPEF inactivates cancer cells metastasis and invasion by inhibiting Wnt/β-Catenin signaling pathway to down-regulate expressions of VEGF and MMPs family proteins *in vitro.* (A) Migration ability of cancer cells exposed to nsPEF was tested by trans-well assay. The migrated cells exposed to nsPEF were stained purple by 0.1% crystal violet solution, observed under light microscope for 40 or 400 magnifications, and counted for statistical analysis. Original magnification, 40× & 400×. ***p<0.001. (B) Invasion ability of cancer cells exposed to nsPEF was detected using matrigel invasion assay. After nsPEF treatment, the cells that possessed invasion ability penetrated through the matrigel, were stained purple by 0.1% crystal violet solution, and were counted for statistical analysis. Original magnification, 40× & 400×. ***p<0.001. (C) Protein expressions of Wnt/β-Catenin signaling pathway including hDPR1, β-Catenin and c-Myc in cancer cells after exposure to nsPEF with different intensities were detected by Western-blot assay. (D) Protein expressions of MMPs family and VEGF in cancer cells after exposure to nsPEF with different intensities were detected by Western-blot assay. Relative intensity of each protein was analyzed by Photoshop CS4 software. *p<0.05, **p<0.01, ***p<0.001.

Activation of metastasis and invasion is an important hallmark of cancer [22,23]. The degree of metastasis and invasion is a standard to classify stage of malignant tumor, and also directly determines therapeutic strategies of cancers and survival of patients [35]. , so we detected the ability of migration and invasion of cancer cells post treatment. In our study, migration ability of cancer cells post pulse was sharply cut down compared with the control by Trans-well assay (p<0.001) (Figure **4A**). Furthermore, we found that cancer cells exposed to nsPEF possessed a significantly weak ability to invade compared to the control using matrigel invasion assay (p<0.001) (Figure **4B**). These results proved that nsPEF could decrease cell metastasis and invasion in cancers.

Accumulated evidence has demonstrated that Wnt/β-Catenin signaling pathway plays a critical role in embryonic development and human various malignancies [36]. The actions of Wnt/β-Catenin signaling pathway on target cells mainly include alterations in gene expression, cell polarization, migration and invasion through regulation of distinct downstream responsive molecules [37]. As downstream effector of Wnt/β-Catenin signaling pathway, MMPs family proteins and VEGF play a critical role in tumor invasion and metastasis [38]. Thus we detected protein expressions of Wnt/β-Catenin signaling pathway, MMPs family and VEGF. We found that expressions of Wnt/β-Catenin signaling pathway proteins mainly including hDPR1, β-Catenin and c-Myc in cancer cells exposed to nsPEF were remarkably decreased with dose-dependence versus the control (p<0.05 at 20kV/cm, p<0.01 at 40kV/cm, p<0.001 at 60kV/cm) (Figure **4C**). Furthermore, results also indicated that expressions of VEGF and MMPs family proteins mainly including MMP1, MMP2, MMP9, MMP11, MMP12, MMP14 and MMP21 were significantly reduced at different degrees with different intensities of nsPEF versus the control (Figure **4D**). So we thought that nsPEF could suppress Wnt/β-Catenin signaling pathway to down-regulate gene expressions of VEGF and MMPs family proteins.

These results strongly suggested that nsPEF could inactivate metastasis and invasion abilities of cancer cells by inhibiting Wnt/β-Catenin signaling pathway to down-regulate gene expressions of VEGF and MMPs family proteins.

### NsPEF functioned safely and effectively as an anti-tumor therapy *in vivo*

On basis of the *in vitro* experiment, nsPEF could induce cell apoptosis, inhibit cell proliferation, and inactivate metastasis and invasion. To further identify the anti-tumor function of nsPEF, we performed nsPEF experiment in the ectopic tumor-bearing mice model. The ectopic tumor model is a pilot study for more in-depth orthotopic model that more closely reflects clinical applications.

**Figure 5:**
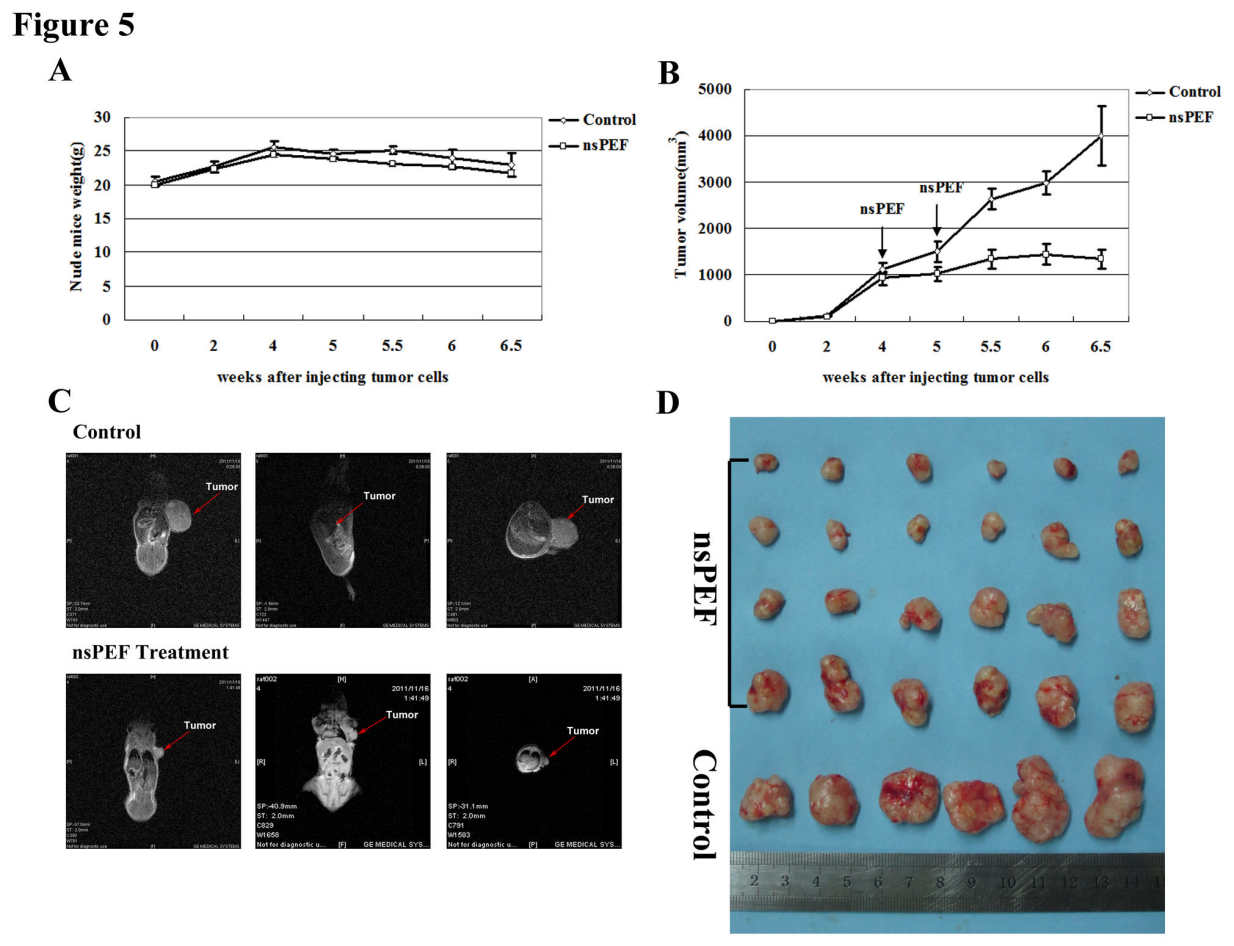
NsPEF functions safely and effectively as an anti-tumor therapy *in* *vivo*. (A) Weights of tumor-bearing mice with or without nsPEF at different time after injecting tumor cells were recorded. (B) Growth curve of tumor with or without nsPEF at different time after injecting tumor cells was shown. (C) Visual comparison of tumor with or without nsPEF through *in* *vivo* imaging of MRI for tumor-bearing mice was performed. (D) Objective comparison of most tumors in most tumor-bearing mice was performed.

During the whole animal experiment, 2 mice in the nsPEF group died from over-dosed anesthesia and 1 in the Control died from ambiguous causes. We observed survival condition of the rest 47 tumor-bearing mice (16 in the Control and 31 in the nsPEF group), and noted that nsPEF maintained mice normal physical activity and had no influence on mice weight (Figure **5A**), indicating that anti-tumor function of nsPEF was safe for the body itself *in vivo*. Meanwhile, we noted a skin scar in the electrode coating location, but it could recover soon within 1-3 days. So we speculated that the scar could be avoided when melanoma was treated or only tumor mass and micro-environment was put within electric fields.

Moreover, we observed tumor growth curve and compared tumor volume with or without nsPEF. We found that tumor significantly became small and tumor growth was remarkably repressed after nsPEF treatment for twice compared with the control. And tumor growth inhibition became gradually larger with prolonged time post pulse (Figure **5B**). To obtain a visual tumor image, we performed the *in vivo* imaging of tumor-bearing mice and found that tumor was much smaller in nsPEF group than that in the control from different angles (Figure **5C**). In addition, all mice were sacrificed on 6.5 weeks after cells injection. We objectively compared all tumors in the mice and found that tumors were significantly small in nsPEF group versus the control (Figure **5D**). In short, *in vivo* experiment revealed that tumor growth was significantly inhibited and tumor obviously became small after nsPEF treatment, which suggested the safe and effective anti-tumor function of nsPEF *in vivo*.

### How nsPEF functioned as an anti-tumor therapy *in vivo*

**Figure 6:**
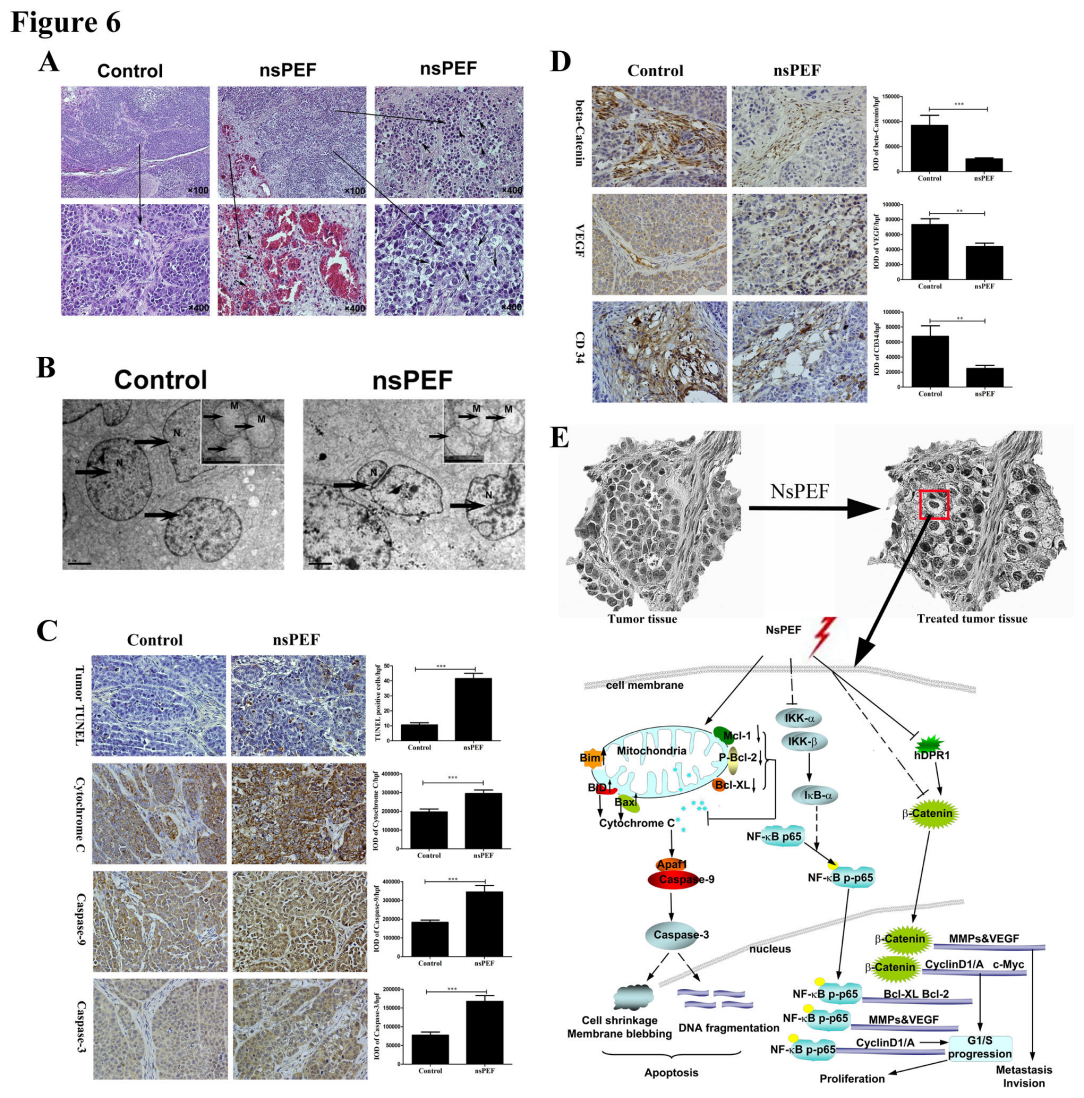
NsPEF induces tumor apoptosis, destroyed tumor microenvironment and depressed angiogenesis in tumor tissue *in vivo*, and its molecular mechanism analysis. (A) Representative tumor histology. Tumor sections were stained with H and E. Original magnification, 100× & 400×. Cell shrinkage, angiorhagia, and leucocytes infiltration were found. (B) Ultrastructure for tumor tissue with or without nsPEF was observed by TEM. N: nuclear changes including nuclear shrinkage and apoptotic body formation. M: mitochondria degeneration. (C) Tumor apoptosis was detected by TUNEL assay, and apoptosis relative proteins including Cytochrome C, Caspase-9 and Caspase-3 were determined by immunohistochemistry staining (IHC). IHC results were analyzed by Image Pro-Plus software. IOD, integrated optical density. hpf, high-powered field. Original magnification, 400×. ***p<0.001. (D) Protein expressions of β-Catenin, VEGF and CD34 were detected by IHC staining. **p<0.01, ***p<0.001. IHC results were analyzed by Image Pro-Plus software. IOD, integrated optical density. hpf, high-powered field. Original magnification, 400×. (E) Molecular mechanism analysis of nsPEF inhibiting cancer growth *in* *vitro* and *in* *vivo*.

To further explore possible mechanisms of anti-tumor function of nsPEF *in vivo*, we next carried out H&E staining, TEM, TUNEL and IHC staining for tumor tissue. First, we observed cell shrinkage, nuclear-cytoplasmic ratio reduction, angiorhagia, substantial erythrocytes distribution and leucocytes infiltration in tumor tissue in nsPEF group versus the control by histology (Figure **6A**). We also noted nuclear shrinkage, chromatin condensation and margination, apoptotic body formation and mitochondria degeneration by tumor TEM in nsPEF group compared with the control (Figure **6B**). Subsequently, we performed TUNEL assay and IHC staining to explore apoptosis degree in tumor. We found that TUNEL positive cells per high-power field (/hpf) were significantly increased in nsPEF group versus the control (p<0.001), and that proteins expressions of Cytochrome-C, Caspase-9 and Caspase-3 in tumor were also even higher in nsPEF group than those in the control by IHC (all p<0.001) (Figure **6C**). In addition, we detected expressions of Wnt/β-Catenin signaling and angiogenesis marker proteins to research tumor micro-environment changes (Figure **6D**). We found that β-Catenin protein in tumor was mainly located in vascular areas in the control but significantly reduced in tumor after nsPEF treatment (p<0.001). Meanwhile, angiogenesis markers including VEGF and CD34 proteins were distributed in the gap between tumor cells and vascular areas in the control but remarkably decreased in nsPEF group (both p<0.001).

These results indicated that nsPEF functioned as an anti-tumor therapy through inducing tumor cell apoptosis, destroying tumor microenvironment, and depressing angiogenesis in tumor *in vivo*.

NF-κB signaling pathway is involved in a variety of cellular functions, including self-sufficiency in growth signals, insensitivity to growth-inhibitors, evasion of apoptosis, limitless replicative potential, tissue invasion and metastasis, and sustained angiogenesis [23,39]. Wnt/β-Catenin signaling pathway probably integrates with other niche-derived signals such as Shh, Notch and BMP [40,41,42]. In some conditions, these signals may function independently for respective aspects of cells or tissue self-renewal, such as survival, proliferation, inhibition of differentiation, metastasis and invasion. In other contexts, the various signaling pathways may function in a shared hierarchy and regulate each other. For example, NF-κB signaling pathway had been proved to regulate expressions of MMPs family proteins and VEGF [43] which also were regulated by Wnt/β-Catenin signaling pathway. Moreover, Wnt/β-Catenin signaling pathway can regulate expression of cyclinD1 in colon carcinoma cells [44] and target identification of c-Myc oncogene in colorectal cancers [45], while NF-κB signaling pathway target genes regulating proliferation include CyclinD1, Cyclin E, CDK2 and c-Myc [33].

With highly compressed power, ultra short pulse durations, rapid rise times and high electric fields, nsPEF can penetrate into cell before plasma membrane is fully charged. Thus, nsPEF can minimally affect plasma membrane but target on subcellular structures, such as mitochondria and nuclear [8]. Alterations of membrane potential of subcellular structures could influence a series of signaling pathways, such as NF-κB [33,46]and Wnt/β-Catenin signaling pathway [36,47], leading to extensive biological effects.

Radiofrequency ablation (RFA) is a widely used thermal procedure that is reported to lead in many cases to incomplete tumor ablation [48,49], which is the result of a cooling or heat sink effect of nearby vessels, so the application of RFA should be considered problematic. However, as a non-thermal therapy, nsPEF can overcome the above disadvantages, which not only targets multiple programmed cell death mechanisms for apoptosis induction [9] and anti-angiogenesis [11], but also presents an apparent broad specificity for cell death induction [7], effective for all cells within electric fields, including rapidly growing tumor cells, slower growing host cells that have been hijacked by tumors and cancer stem cells, all constituting the tumor mass and the microenvironment. Furthermore, nsPEF treatment can destroy tumor small vessel to induce local infarction [11], which deprives tumors of feeder vessels that are important for immediate oxygenation and nutrition, and also have the potential for enhancing immune surveillance from cells undergoing apoptotic.

## Conclusions

In this study, we explored the possible molecular mechanisms of nsPEF on pancreatic carcinoma cells from the major hallmarks of cancer including resisting apoptosis, enabling replicative immortality, activating invasion and metastasis *in vitro*. Meanwhile, we established HCC tumor-bearing mice model to observe the safety and effectiveness of nsPEF tumor ablation, and investigated how nsPEF functioned as an anti-tumor therapy *in vivo*. We found that nsPEF has extensive biological effects in cancers, and clarified its possible molecular mechanisms, as illustrated in Figure **6E**. It cannot only induce apoptosis in cancers cells via dependent-mitochondria intrinsic apoptosis pathway, but also inhibit cell proliferation through repressing NF-κB signaling pathway to reduce expressions of Cyclin proteins. Moreover, nsPEF can also inactivate metastasis and invasion in cancer cells by suppressing Wnt/β-Catenin signaling pathway to down-regulating expressions of VEGF and MMPs family proteins. More importantly, nsPEF can function safely and effectively as an anti-cancer therapy through inducing tumor cell apoptosis, destroying tumor microenvironment and depressing angiogenesis in tumor *in vivo*. These findings may provide a creative and effective therapeutic strategy for cancers.

## Supporting Information

Figure S1 **Equipments of nsPEF generator for cancer cells and tumor tissue.** (A) The whole equipment of nsPEF generator includes a power supply, a digital phosphor oscilloscope, a high voltage probe and a generation apparatus of nsPEF. (B) Typical waveforms of nsPEF. (C) Treatment apparatus of nsPEF for cancer cells. (D) Power supply of nsPEF for tumor tissue. (E) Operation of nsPEF for tumor tissue.
(TIF)

Table S1 **Primary antibodies and details of Western blotting and IHC.**
(DOC)
