# Supplementary figures and images for: Nanosecond Pulsed Electric Field Inhibits Cancer Growth Followed by Alteration in Expressions of NF-κB and Wnt/β-Catenin Signaling Molecules
Source: PLoS One. 2013 Sep 17;8(9):e74322. doi: 10.1371/journal.pone.0074322 (PMC3775773; doi:10.1371/journal.pone.0074322)

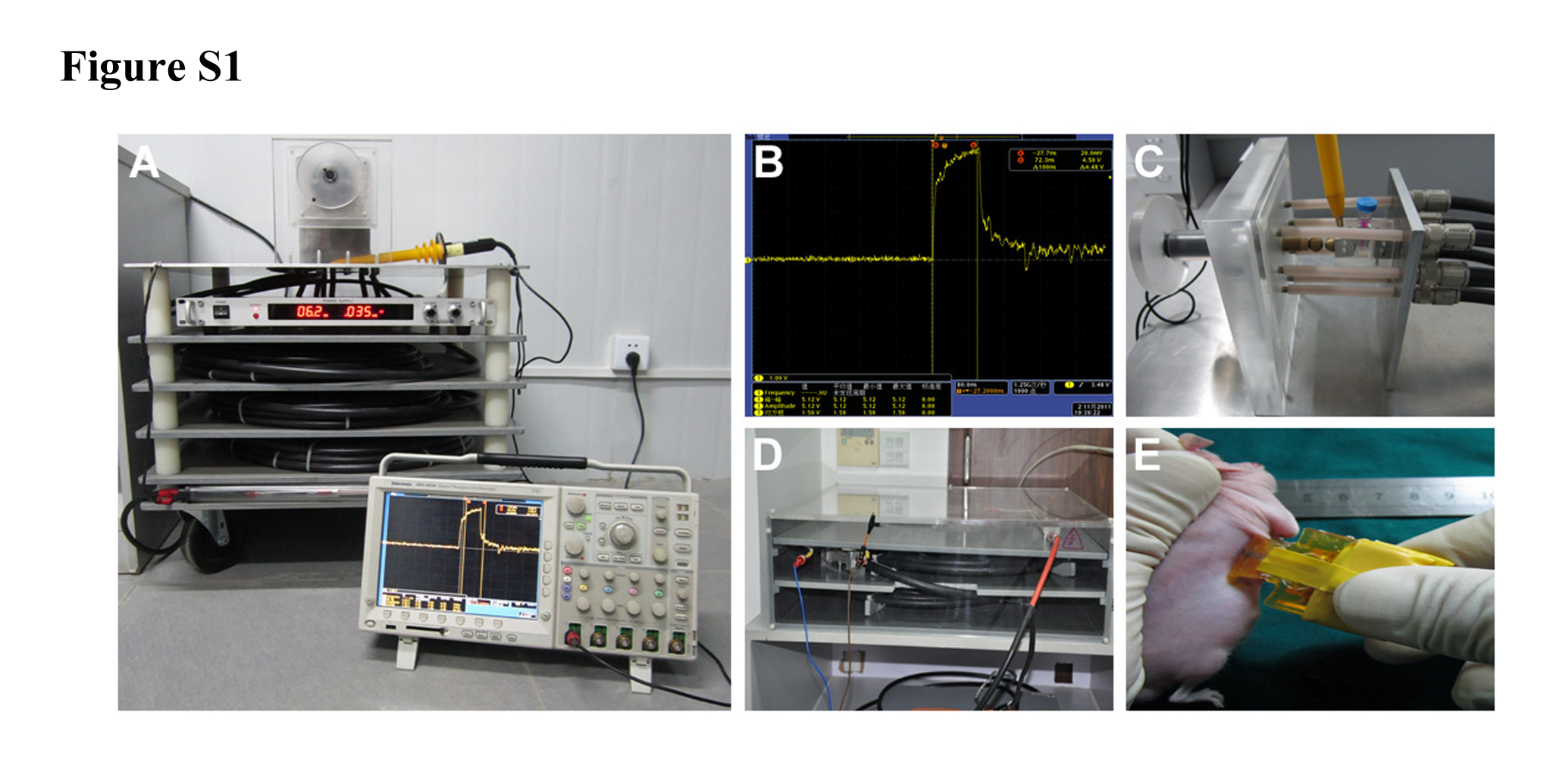

Supplement: Figure S1 — Equipments of nsPEF generator for cancer cells and tumor tissue. (A) The whole equipment of nsPEF generator includes a power supply, a digital phosphor oscilloscope, a high voltage probe and a generation apparatus of nsPEF. (B) Typical waveforms of nsPEF. (C) Treatment apparatus of nsPEF for cancer cells. (D) Power supply of nsPEF for tumor tissue. (E) Operation of nsPEF for tumor tissue. (TIF) [file pone.0074322.s001.tif]
