# Supplementary material for: Nanosecond Pulsed Electric Field Inhibits Cancer Growth Followed by Alteration in Expressions of NF-κB and Wnt/β-Catenin Signaling Molecules
Source: PLoS One. 2013 Sep 17;8(9):e74322. doi: 10.1371/journal.pone.0074322 (PMC3775773; doi:10.1371/journal.pone.0074322)
Supplement: Table S1 — Primary antibodies and details of Western blotting and IHC. (DOC) [file pone.0074322.s002.doc]

**Table S1 Primary antibodies and details of Western blotting** and IHC

| Primary antibodies | Company | Article Number |
| --- | --- | --- |
| Pro-Survival Bcl-2 Family Antibody Sampler Kit | Cell Signaling Technology | #9941 |
| Pro-Apoptosis Bcl-2 Family Antibody Sampler Kit | Cell Signaling Technology | #9942 |
| Cytochrome C Antibody | Abcam Ltd., HongKong | ab13575 |
| Caspase-3 Antibody | Abcam Ltd., HongKong | ab136812 |
| Caspase-9 Antibody | Abcam Ltd., HongKong | ab32539 |
| [IKKα Antibody](http://www.cellsignal.com/products/2682.html) | Cell Signaling Technology | #2682 |
| IKK-β Antibody | Cell Signaling Technology | #2370 |
| p-IKK-α/β Antibody | Cell Signaling Technology | #2697 |
| IκB-α Antibody | Cell Signaling Technology | #4814 |
| NF-κB p65 Antibody | Cell Signaling Technology | #8242 |
| NF-κB p-p65 Antibody | Cell Signaling Technology | #3033 |
| hDPR1 Antibody | Abcam Ltd., HongKong | ab51260 |
| β-Catenin Antibody | Cell Signaling Technology | #9562 |
| c-Myc Antibody | Cell Signaling Technology | #5605 |
| Cyclin A Antibody | Cell Signaling Technology | #4656 |
| Cyclin D1 Antibody | Cell Signaling Technology | #2978 |
| Cyclin E Antibody | Cell Signaling Technology | #4129 |
| MMPs Family Kit | Epitomics | 5006-1 |
| VEGF Antibody | Epitomics | 1909-1 |
| CD34 Antibody | Epitomics | 2150-1 |
